# Anti-tumour effect of neo-antigen-reactive T cells induced by RNA mutanome vaccine in mouse lung cancer

**DOI:** 10.1007/s00432-021-03735-y

**Published:** 2021-07-21

**Authors:** Jiaxing Sun, Jing Zhang, Haiyan Hu, Huan Qin, Ximing Liao, Feilong Wang, Wei Zhang, Qi Yin, Xiaoping Su, Yanan He, Wenfeng Li, Kun Wang, Qiang Li

**Affiliations:** 1 Department of Pulmonary and Critical Care Medicine, Shanghai East Hospital, Tongji University School of Medicine, Shanghai, China; 2 Center of Diagnosis and Treatment of Breast Disease, The Affiliated Hospital of Qingdao University, Qingdao, China; 3 Department of Pulmonary and Critical Care Medicine, Shanghai Changhai Hospital, Second Military Medical University, Shanghai, China; 4 School of Basic Medicine, Wenzhou Medical University, Wenzhou Tea Mountain Higher Education Park, Wenzhou, China; 5 Department of Chemoradiation Oncology, The First Affiliated Hospital of Wenzhou Medical University, Wenzhou, Zhejiang China

**Keywords:** Lung cancer, Neoantigens, Neoantigen-reactive T cells, Adoptive cell therapy, Immunotherapy, Vaccine

## Abstract

**Purpose:**

Mutation-specific T-cell response to epithelial cancers and T-cell-based immunotherapy has been successfully used to treat several human solid cancers. We aimed to investigate the anti-tumour effect of neo-antigen-reactive T(NRT) cells induced by RNA mutanome vaccine, which may serve as a feasible and effective therapeutic approach for lung cancer.

**Methods:**

We predicted candidate neo-antigens according to the mutant gene analysis by sequencing the mouse Lewis cells and C57BL/6 mouse tail tissue. RNA vaccine was prepared with the neo-antigens as the template. We assessed antitumor efficacy, cytokine secretion and pathological changes after adoptive transfer of NRT cells in vitro and vivo experiments.

**Results:**

We identified 10 non-synonymous somatic mutations and successfully generated NRT cells. The percentage of T-cell activation proportion was increased from 0.072% in conventional T cells to 9.96% in NRT cells. Interferon-γ secretion augmented from 17.8 to 24.2% as well. As an in vivo model, adoptive NRT cell infusion could promote active T-cell infiltration into the tumour tissue and could delay tumour progression.

**Conclusion:**

NRT cells induced by RNA mutanome vaccine exert a significant anti-tumour effect in mouse lung cancer, and adoptive NRT cell therapy might be considered a feasible, effective therapeutic approach for lung cancer.

**Supplementary Information:**

The online version contains supplementary material available at 10.1007/s00432-021-03735-y.

## Introduction

Lung cancer is the leading cause of cancer death worldwide. Nearly 25% of all cancer deaths are due to lung cancer, and approximately 82% of deaths are related to smoking (Siegel et al. 2021). For non-small cell lung cancer (NSCLC), surgical removal of cancer in early stages of the disease prolongs survival of patients; however, the overall 5-year survival rate is only 15% (Siegel et al. 2016). Patients in advanced stage of the disease are less prone to benefit from surgical interventions or systemic treatments. Cancer Statistics revealed that the 5-year survival rate of patients with advanced NSCLC is merely 6% (Siegel et al. 2021). However, in recent years, targeted therapeutic drugs and immune checkpoint inhibitors (ICIs) have significantly improved the disease prognosis in patients in clinical trials and have been incorporated in the standard management of advanced stage NSCLC (Guibert and Mazières 2015; Reck et al. 2016; Soria et al. 2018). Immune checkpoints are the surface proteins present on T cells and other immune cells and act as negative regulators of immune activation by tumour antigens. Immunomodulatory drugs disrupt this negative signalling between immune cells (predominantly T cells) and tumour cells by removing the brake on T-cell activation by antigen-presenting cells (APCs) (Seebacher et al. 2019). Monoclonal antibodies targeting programmed cell death protein 1 (PD-1) (e.g., nivolumab, pembrolizumab) and programmed death-ligand 1 (PD-L1) (e.g., atezolizumab) have demonstrated significant improvements in progression-free survival (PFS) and overall survival in the latest clinical trials conducted in patients in the advanced stage of NSCLC (Borghaei et al. 2015; Brahmer et al. 2015; Reck et al. 2016; Rittmeyer et al. 2017). However, ICIs may also promote the attack of T cells on self-antigens, which clinically manifests as a spectrum of diseases including pneumonitis, dermatitis, thyroiditis, hepatitis, colitis and inflammatory arthritis, which are collectively known as immune-related adverse events (irAEs) (Rittmeyer et al. 2017). Among all irAEs, checkpoint inhibitor pneumonitis (CIP) occurs in approximately 3–5% of patients receiving ICIs, although the real-world incidence of CIP may be higher. Moreover, patients with CIP who do not improve with steroids have higher mortality (Suresh et al. 2018). Therefore, exploring an effective and low-toxic treatment strategy for improving the survival rate and the quality of life of patients with NSCLC remains a challenge for researchers.

Studies pertaining to cancer immunotherapies, such as adoptive T-cell therapy (ACT) and chimeric antigen receptor (CAR) therapy, have indicated that the anti-tumour responses of T cells can be stimulated by recognising mutated neo-antigens (Dudley et al. 2002; Fry et al. 2018; Tran et al. 2014). Tumour neo-antigens are the repertoire of peptides expressed on the surface of tumour cells but are not expressed in normal mammalian tissues and are recognised by antigen-specific T-cell receptors (TCRs) through the cooperation of major histocompatibility complex (MHC) molecules (Schumacher and Schreiber 2015). Numerous non-synonymous genetic alterations, such as gene fusions, insertions and deletions, frameshift mutations, structural variants, and single-nucleotide variants (SNVs), can induce the expression of neo-antigens (Jiang et al. 2019). Following melanoma, lung cancer possesses the highest mutation burden and thus expresses mutated neo-antigenic peptides (Lawrence et al. 2013), which can trigger T-cell responses and can be targeted effectively by T cells. High tumour specificity and immunogenicity of neo-antigens make them an ideal candidate for ACT (Kilic et al. 2011). Immunotherapy based on ACT of highly selected tumour-infiltrating lymphocytes (TILs) (referred to as TIL-ACT) shows tumour regression in patients with metastatic melanoma, as reported by Rosenberg in 2002 (Dudley et al. 2002). In 2014, Professor Steve Rosenberg et al. reported the case of a patient with metastatic cholangiocarcinoma who received TIL-ACT containing approximately 25% mutation-specific poly-functional TH1 cells, which could recognise a mutation in erbb2-interacting protein expressed by cancer cells, resulting in prolonged stabilisation of the disease (Tran et al. 2014). In 2016, researchers reported that the use of the aforementioned method in a patient with metastatic colorectal cancer produced a good curative effect (Tran et al. 2016). In 2019, Professor Fangjun Chen described the adoptive therapy based on tumour NRT cells through which several patients with advanced cancer were successfully treated. A patient with metastatic thymoma demonstrated a complete and durable response to a combination of personalised tumour vaccine and NRT cell refusion 29 months after the treatment. Immune-related partial response was observed in another patient with metastatic pancreatic cancer treated with a combination of personalised tumour vaccine and NRT cell refusion; prolonged stabilisation of the disease, with a median PFS of 8.6 months, was achieved in 4 patients (Chen et al. 2019). Although clinical success in melanoma and other solid tumours has been achieved by isolating and amplifying TILs on the basis of neo-antigen–RNA vaccines, none of the studies have focussed on the preparation of NRT cells based on neo-antigen–RNA vaccines.

In the present study, we aimed to investigate the anti-tumour effect of NRT cells induced by RNA mutanome vaccine. We identified 10 non-synonymous somatic mutations from Lewis cells and C57BL/6 mouse tail tissue and generated a neoantigen–RNA vaccine. Our results demonstrate that lung cancer neo-antigens can be recognised by the immune system and that the adoptive transfer of NRT cells based on RNA mutanome vaccine exerts a significant anti-tumour effect in mouse lung cancer.

## Materials and methods

### Materials

Reagents and antibodies used in this study are listed in Supplementary Table 1.

### Cell culture

Lewis cell lines were obtained from the American type culture collection. The cells were cultured in RPMI1640 medium supplemented with 10% foetal bovine serum (FBS), 100 U/mL penicillin, and 100 µg/mL streptomycin at 37 °C and in the presence of 5% CO_2_.

### Mice

Six- to eight-week-old sex-matched C57BL/6 mice were purchased from Vital River Laboratory Animal Technology Co. Ltd. Beijing, China. All animal experiments were performed under specific pathogen-free conditions. The animal experiments were approved by the Ethics Committee of East Hospital, Shanghai, China.

### Tumour-bearing mouse model

To generate conventional T cells, we injected 2 × 10^6^ Lewis cells into the rear flank of 6- to 8-week-old sex-matched C57BL/6 mice. For the ACT experiment, 5 × 10^5^ Lewis cells were injected into the rear flank of the C57BL/6 mice. The mice were monitored daily, and the tumour volumes were measured every other day as tumour could be palpated subcutaneously. The tumour growth was monitored through caliper measurements, and the volume was calculated using the following formula: tumour volume (mm^3^) = *D* × *d*^2^/2, where *D* and *d* indicate the longest and the shortest diameters, respectively.

### Generation of dendritic cells

To acquire bone marrow-derived dendritic cells (BMDCs), 6- to 8-week-old sex-matched C57BL/6 mice were euthanised based on the local animal care guidelines. BMDCs were generated according to a previously described method (Roney 2019). Briefly, bone marrow-derived cells were cultured in complete RPMI (RPMI-1640, 55 µM 2-mercaptoethanol, 10 mM of HEPES, 100 U/mL of penicillin, 100 μg/mL of streptomycin sulphate, and 10% of heat-inactivated FBS) supplemented with 20 ng/mL of granulocyte–macrophage colony-stimulating factor (GM–CSF) during the first 3 days and in the same medium supplemented with 10 ng/mL of GM–CSF during the following 7 days at 37 °C and 5% CO_2_. The complete RPMI was supplemented with 10 ng/mL of interleukin (IL)-4 from the sixth day to the last day. On day 10, BMDCs were harvested, which were floating or lightly adherent in the culture.

### T-cell preparation

Tumour could be palpated subcutaneously 10 days after inoculation of Lewis cells. The fresh spleen was obtained by euthanising the mouse based on the local animal care guidelines. T cells were sorted using the Pan T Cell Isolation Kit II in the super clean platform and were frozen in liquid nitrogen. Briefly, a single cell suspension was prepared from the spleen of tumour-bearing mice using Miltenyi gentle MACS™ Dissociator. Then, splenic single cells were isolated through negative selection using magnetic beads on Miltenyi Manual Separators to acquire T cells. The acquired T cells had more than 93% purity. The conventional T cells were prepared using the same method.

### Tissue sequencing and neo-antigen prediction

For potential neoantigen profiling, the Lewis cells and C57BL/6 mouse tail tissue were extracted, and whole-exome sequencing (WES), RNA sequencing with coverage depths of 200 × , and transcriptomic sequencing were performed. Briefly, exome and transcriptome libraries were constructed, according to manufacturer’s instructions, following DNA/RNA extraction from Lewis cells and DNA extraction from the mouse tail tissue (Castle et al. 2012). The libraries were then sequenced using the Illumina Novaseq 6000 platform (paired end, 150 bp). Sequencing and epitope prediction were performed according to a previous study (Zeng et al. 2020). For mutation detection, DNA reads were compared with the reference genome mm10 using BWA (Li and Durbin 2009). Duplicate exomes from the Lewis cells and mouse tail tissue were analysed for the presence of SNVs. The sites were identified and screened for homozygous genotypes in normal samples, thus maintaining a high degree of specificity in SNV. The remaining sites were further examined to determine the presumed homozygous or heterozygous mutation event. The suspicious sites were screened to exclude potential false positives, test the sum of duplicates, and merge the duplicates. We compared the genomic coordinates of the identified variants with the detailed known gene transcription coordinates in University of California-Santa Cruz (UCSC) and further determined the relationship between the mutations and genes, transcription, expression values derived from RNA-Seq, and changes in potential amino acid sequences. For RNA-Seq, RNA reads were compared with the mm10 reference genome and transcriptome using Bowtie (Ghosh and Chan 2016). We measured the gene expression by comparing known gene transcripts with the detailed exon coordinates in UCSC and then normalised to RPKM units (Chen et al. 2019).

Immunogenicity of the remaining mutations was then evaluated using the MuPeXI pipeline (Bjerregaard et al. 2017). First, 8–11 and 12–15 amino acid-long mutant peptides were generated for MHCI- and MHC II-restricted neoantigen prediction, respectively. The expression of mutant genes was measured using RNA-Seq data in transcripts per million. The mutant allele frequency was determined using variant caller MuTect2 (Kim et al. 2019). Each peptide was provided a priority score on the basis of HLA-binding affinity, expression level, similarity to self-peptides, and mutant allele frequency. Peptides with a priority score of > 0 were selected as neoantigen candidates.

### Recombinant minigenes-pcDNA3.1 plasmids

Tandem mini-genes (TMGs) were constructed as described previously (Tran et al. 2014). Mini-genes were constructed for each non-synonymous substitution mutation. We encoded the mutated amino acid and surrounding upstream and downstream native amino acids of the wild-type protein sequence for a total length of 27 amino acids. Multiple minigenes were arranged in tandem without additional linker sequences and were synthesised [Sangon Biotech (Shanghai) Co. Ltd]. The TMG was inserted into the pcDNA3.1 vector using available EcoRI and BamHI cut sites, namely minigene-pcDNA3.1 plasmids. The aforementioned procedure was performed according to the manufacturer’s instructions.

### Generation of in vitro transcribed RNA

Plasmids encoding the minigenes were linearised using the restriction enzyme Fast Digest xhol. Approximately 1 µg of linearised plasmid was used for the preparation of in vitro transcribed (IVT) RNA using the Mmessage mmachine T7 Ultra Kit, according to the manufacturer’s instructions. RNA was precipitated and purified using the RNeasy^®^ Plus Mini Kit. RNA purity and levels were measured using the NanoDrop spectrophotometer. The A260:A280 ratio should be at least 1.8. RNA was aliquoted into microtubes and stored at − 80 °C until use.

### Transfection of dendritic cells with IVT RNA

Dendritic cells (DCs) were transfected with IVT RNA using Amaxa^®^ Mouse Dendritic Cell Nucleofector^®^ Kit, according to the manufacturer’s instructions. Briefly, for a single reaction, we used 82 µL of Nucleofector^®^ Solution and 18 µL of supplement to make a total reaction volume of 100 µL. A total of 1–2.5 × 10^6^ DCs and 1 µg of IVT RNA were re-suspended in the 100 µL reaction volume. Cell/RNA suspension was transferred into a certified cuvette (sample covered the bottom of the cuvette without air bubbles). Appropriate Nucleofector^®^ Program Y-001 was selected until the programme was finished. Following electroporation, DCs were immediately transferred to DC medium supplemented with mouse GM-CSF and IL-4. IVT RNA-transfected DCs (henceforth referred to as Neo-DCs) were incubated overnight at 37 °C in the presence of 5% CO_2_.

### Quantitative polymerase chain reaction for Neo-dendritic cells

Total RNA was extracted from Neo-DCs and conventional DCs using the RNeasy^®^ Plus Mini Kit and then reverse transcribed to complementary DNA (cDNA) using cDNA Supermix. Minigene-specific primer (forward: TTCAGCAGCTCAGCCACC; reverse: AGCAACAATGCCCACGAT) and primer for β-actin (forward: GGTCCACACCCGCCACCAG; reverse: CACATGCCGGAGCCGTTGTC) were purchased from Life Technologies. Data were normalised to β-actin expression.

### Generation of neoantigen-reactive T cells

Neo-DCs were incubated with T cells in a ratio of 1:10 in complete RPMI Medium 1640 supplemented with 10% FBS, 100 U/mL penicillin, 100 µg/mL streptomycin, and 10 mM HEPES at 37 °C and in the presence of 5% CO_2_ for 8 h. Then, 100 U/mL of IL-2 was added to the DC–T cell suspension for another 48 h under similar conditions. From day 3, 30 ng/mL of OKT3 (InVivoMAb anti-mouse CD3ε) was added to the fresh complete RPMI medium containing 6000 U/mL of IL-2 and 20 ng/mL of IL-7. Depending on the growth of NRT cells, fresh Neo-DCs and OKT3 antibody were co-cultured with NRT cells for another 2-time re-stimulation. During day 10–day 14, NRT cells were harvested and re-suspended with phosphate-buffered saline (PBS). Before cell transplantation, the phenotype and interferon (IFN)-γ release assay were performed using flow cytometry and enzyme-linked immunospot (ELISpot) assay.

### Interferon-γ ELIspot assay

IFN-γ secretion by NRT cells was measured through the ELISpot assay using IFN-γ ELISpot- plus kit, according to the manufacturer’s instructions. Briefly, after the preparation of ELISpot plate, 2.5 × 10^5^ conventional T cells or NRT cells were co-cultured with 2.5 × 10^4^ conventional DCs or Neo-DCs (200 µL/well) in the plate for 24 h without exogenously added cytokines. The cells were removed by emptying the plate and washing 5 times with PBS and subsequently incubated with detection antibody (R4-6A2-biotin, 1 µg/mL) containing foetal calf serum (PBS + 0.5%FBS) for 2 hat room temperature. After washing the plate, streptavidin–ALP (1:1000 dilution, 100 µL/well) was added to the plate and incubated for 1 h at room temperature. Substrate solution (BCIP/NBT-plus, 100 µL/well) was added to each well and incubated until the emergence of distinct spots. Then, deionised water was added to stop colour development. Finally, the spots were imaged and analysed. Spots larger than twice the spots in the control group were considered positive for T-cell reactivity.

### The interferon-γ and tumour necrosis factor-α release assay

The anti-tumour reactivity of NRT cells was determined through the IFN-γ and TNF-α release assay. NRT or conventional T cells were co-cultured overnight with the Lewis cells in different ratios as indicated in a 96-well plate. The cells were centrifuged, supernatant was collected, and the level of secreted IFN-γ and TNF-α was determined through ELISA, according to the manufacturer’s instructions (BioLegend, San Diego, CA). Measurements were performed in triplicates.

To determine intracellular IFN-γ levels, 1 × 10^6^ NRT or conventional T cells were stimulated with the Cell Activation Cocktail for 2 h, according to the manufacturer’s instructions. Flow cytometry was performed by the addition of CD3 antibody (APC conjugated), followed by fixation, permeabilisation, and addition of IFN-γ antibody. The samples were analysed using FlowJo software. Measurements were performed in triplicates.

### Flow cytometry

The anti-mouse monoclonal antibodies used for cell surface staining were CD3-APC and CD137-PE (clone:41BB). All the antibodies were obtained from BioLegend (San Diego, CA). Briefly, the cell pellet was washed with FACS buffer and then incubated with the anti-mouse CD16/32 for 10 min at room temperature to block FC receptors. The surface antibodies were stained in the dark for approximately 20 min. To determine the intracellular IFN-γ levels, 1–2 × 10^6^ NRT/T cells were stimulated using the aforementioned procedure. After stimulation, LIVE/DEAD fixable dye was used to exclude dead cells before blocking of the FC receptors and cell surface staining. Then, the cells were fixed and permeabilised using a Fixation/Permeabilization Kit, followed by staining with an anti-mouse IFN-γ antibody. The cells were washed twice with FACS staining buffer or Perm/Wash™ buffer prior to acquisition on an Arial II-Optics flow cytometer. All the data were gated on live and single cells. The data were analysed using FlowJo software. Measurements were performed in triplicates.

For enrichment of single tumour cell suspensions from induced mouse tumour tissue, tumours were cut into small pieces and digested with Tumor Dissociation Kit (Miltenyi Biotec) followed by erythrocyte lysis. The following surface fluorochrome-conjugated antibodies were purchased from BD Pharmingen/Horizon™: anti-CD4 (Percpcy5.5 or BV421), anti-CD8 (Fitc), anti-CD45 (Fitc), anti-CD11b (Percpcy5.5), anti-F4/80 (PE). Intracellular Foxp3 staining was performed with the anti-mouse Foxp3 (APC) staining set (eBioscience) according to the instructions of the manufacturer. Dead cells were excluded by Fixable Viability Stain 780 or 520 (APC-CY7 or Fitc, BD). The staining and analysing protocol was the same as aforementioned procedure.

### Antitumour effects of neoantigen-reactive T cells in vivo

A total of 5 × 10^5^ Lewis cells were subcutaneously injected into the rear flank of the C57BL/6 mice. When the average tumour volume reached approximately 50 mm^3^, all the mice were pre-treated with cyclophosphamide (CTX, 100 mg/kg) for lympho-depletion (Day 1). A total of 2 × 10^7^ NRT or conventional T cells were injected into the tumour-bearing mice through tail vein (Day 0), and rhIL-2 (180,000 units) was injected intraperitoneally for 3 consecutive days (Day 1–3). The tumour growth was monitored through caliper measurements according to the aforementioned methods. Mice were sacrificed when the tumour exceeded 20 mm in any one dimension or showed signs of suffering, such as the tumour burden > 10% body weight, ulceration, necrosis or infection, and interference with eating or impaired ambulation. Survival of the mice was also assessed. The tumour-bearing mice were sacrificed on day 21. The peripheral blood, tumour tissue, lung, liver and intestine of each mice were harvested for the cytometric bead array (CBA) assay, haematoxylin and eosin (H&E) staining, and immunofluorescence analysis.

### CBA assay for the determination of inflammatory cytokine levels

BD™ CBA Mouse Inflammation Kit was used to quantitatively measure the protein levels of IL-6, IL-10, monocyte chemo-attractant protein 1 (MCP-1), IFN-γ, tumour necrosis factor (TNF), and IL-12p70 in a single serum sample according to the manufacturer’s instructions. Briefly, we mixed the chemokine capture beads with the recombinant standards and mouse serum samples and incubated them with the PE-conjugated detection antibodies to form sandwich complexes. These complexes were analysed using flow cytometry to identify particles with fluorescence characteristics of both the bead and detector. The intensity of PE fluorescence of each sandwich complex revealed the level of the respective cytokine. FCAP Array™ software was used to generate results in the graphical and tabular format.

### Immunofluorescence analysis and histopathological evaluation

Tumour masses from mice were perfused with 0.1 M PBS (pH 7.4), embedded into optimal cutting temperature compound and frozen for cryostat section. Cryostat sections were fixed with 4% PFA for 15 min at room temperature. After PBS washing, cryostat sections were incubated in the blocking solution (PBS containing 3% donkey serum, 1% BSA, 0.3% Triton X-100 at pH 7.4) for 30 min at room temperature. In antibody reaction buffer (PBS containing 1% BSA, 0.3% Triton X-100 at pH 7.4), the samples were stained with primary antibodies overnight at 4 °C. The primary antibodies used for immunofluorescence analysis were: anti-CD3 antibody and anti-CD137 antibody. Cy3-conjugated Goat anti-Rabbit IgG and FITC-conjugated Goat anti-Rabbit IgG were used as the secondary antibodies. For the staining of the nucleus, 4′,6-Diamidino-2-phenylindole (DAPI) was used. After incubation with fluorescent-labelled secondary antibody and DAPI, immunofluorescent microscopic images were obtained and viewed using ECLIPSE C1 Ortho-Fluorescent Microscopy (Nikon, Japan). DAPI emits blue light by absorbing light at the UV excitation wavelength of 330–380 nm and emission wavelength of 420 nm. FITC emits green light at the excitation wavelength of 465–495 nm and emission wavelength of 515–555 nm. CY3 emits red light at the excitation wavelength of 510–560 nm and emission wave length of 590 nm.

For histopathological examination, tumour samples were fixed with neutrally buffered 3.5% formaldehyde and subjected to H&E staining at the Pathology Institute of Service bio technology Co. Ltd. (Wuhan, China). The microscopic evaluation of H&E stained images was performed using DS-U3 (Nikon, Japan).

### Statistical analysis

Statistical analyses were performed using the paired Student’s *t *test in Prism software (GraphPad, San Diego, CA, USA). Data are represented as the mean ± standard error of mean. Values of *P < 0.05, **P < 0.01, ***P < 0.001, and ****P < 0.0001 were considered statistically significant.

## Results

### Neoantigen prediction

Melanoma has become a hotspot in tumour immunology research because it often gives rise to endogenous T cells with antitumour activity. A large number of neoantigen-reactive T cells and the immunoreaction to the unique mutations by TILs may be the underlying reasons (Lawrence et al. 2013; Robbins et al. 2013). After melanoma development, lung cancer possesses the highest mutation burden and thus expresses mutated neoantigentic peptides (Lawrence et al. 2013) that can be targeted effectively by T cells, making them an ideal candidate for the adoptive transfer of NRT cells (Kilic et al. 2011). Thus, neoantigen prediction and generation of IVT RNA became particularly important in our study.

**Fig. 1:**
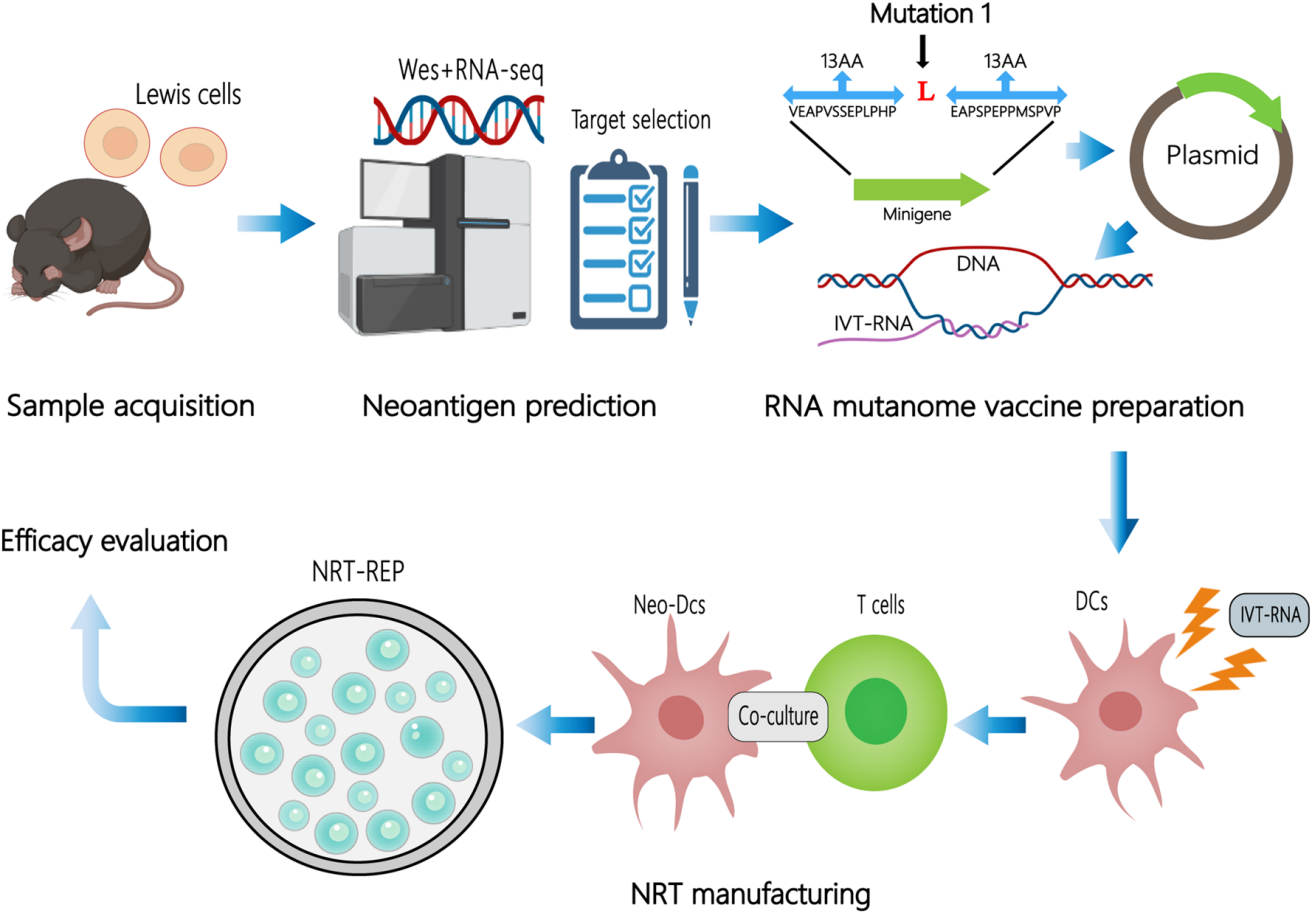
The scheme of the study

According to the scheme of the study, as illustratedin Fig. 1, we performed WES and RNA sequencing of Lewis cells and C57BL/6 mouse tail tissue. Protein-coding variants were ranked by variant allele frequency and mRNA expression. Based on the affinity analysis of the mutation by NetMHCpan V.4.1, 10 mutations with the highest comprehensive score were selected for potentially immunogenic somatic point mutations and TMG synthesis (Table 1).

**Table 1:**
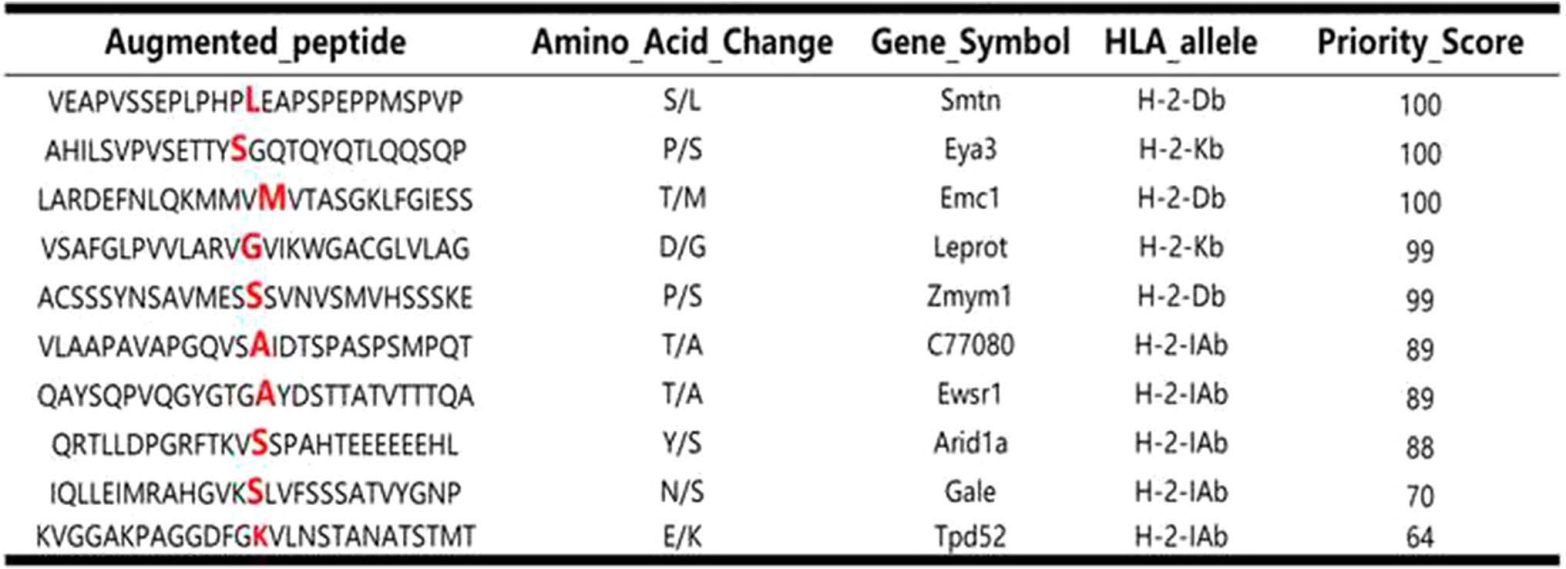
Overview of identified immunogenic somatic point mutations

### Preparation of neoantigen-reactive T cells

**Fig. 2:**
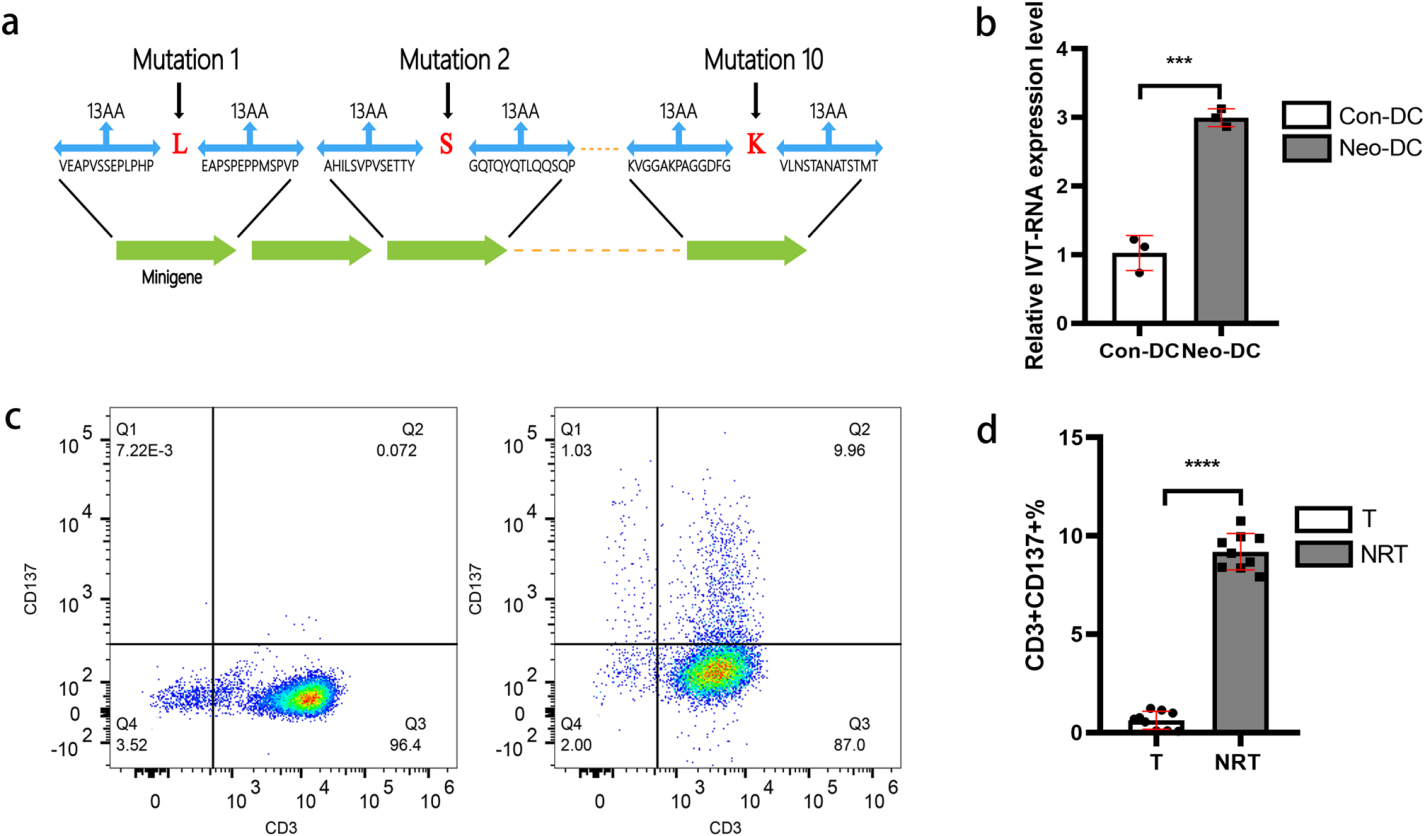
Preparation of NRT cells. **a** The basic structure of the TMG Construct. A variable number of minigenes were genetically fused together to constitutethe TMG. Each minigene encodes for the mutation surrounding for a total length of 27 amino acids. **b** Total RNA was extracted from Neo-DCs and conventional DCs (Con-DC). The expression for IVT RNA was measured using quantitative PCR (*n* = 3). Data were normalised to β-actin expression. Data shown are representative of one of three independent experiments. **c** and **d** T cells were collected after co-culturing with DCs for 48 h. Activated T-cell percentage (CD3^+^/CD137^+^T-cell population) in NRT cell preparation. *T* conventional T-cell group, *NRT* neoantigen-reactive T-cell group. ***P < 0.001, ****P < 0.0001. Data are gated on live cells and further gated on single cells

A variable number of minigenes were genetically fused together to constitute the TMG that encoded for the mutated amino acid, which were then used as the template for generating IVT RNA (Fig. 2a). TMG was inserted into a pcDNA3.1 vector to construct minigene-pcDNA3.1 plasmids, which were used for generating IVT RNA. Then, the transfection of DCs with IVT RNA was performed according to the aforementioned method. To determine the efficacy of DCs transfected with IVT RNA, quantitative PCR was performed, which demonstrated that neoantigen IVT RNA was significantly upregulated in Neo-DCs compared with that in conventional DCs (Fig. 2b). Thus, the manufacturing of Neo-DCs was feasible in our experiment.

T cells were obtained from the Lewis tumour-bearing mouse according to the aforementioned method and the purity of T (CD3^+^T) cells was more than 93% (Fig. 2c). To evaluate the number of activated T cells (NRT cells) after co-culturing with Neo-DCs for 48 h, we analysed the CD3^+^/CD137^+^T cell percentage through flow cytometry. CD137 is a co-stimulatory marker that is expressed on activated T cells and represents the specific interaction of T cells with their target cells. The expression of CD137 on T cells was used to identify and isolate tumour-specific T cells from PBLs and TILs (Seliktar-Ofir et al. 2017; Wolfl et al. 2007). Similarly, the clinical effects of ACT rely on the presence of tumour-reactive T-cell populations (Krishna et al. 2020). As expected, the CD3^+^/CD137^+^ T cell percentage was increased from 0.072% in conventional T cells to 9.96% in NRT cells (Fig. 2c, d), suggesting an increase in the number of NRT cells in the total infusion of T cells and their potential antitumour effect.

### Immune response assessment of NRT cells in vitro

**Fig. 3:**
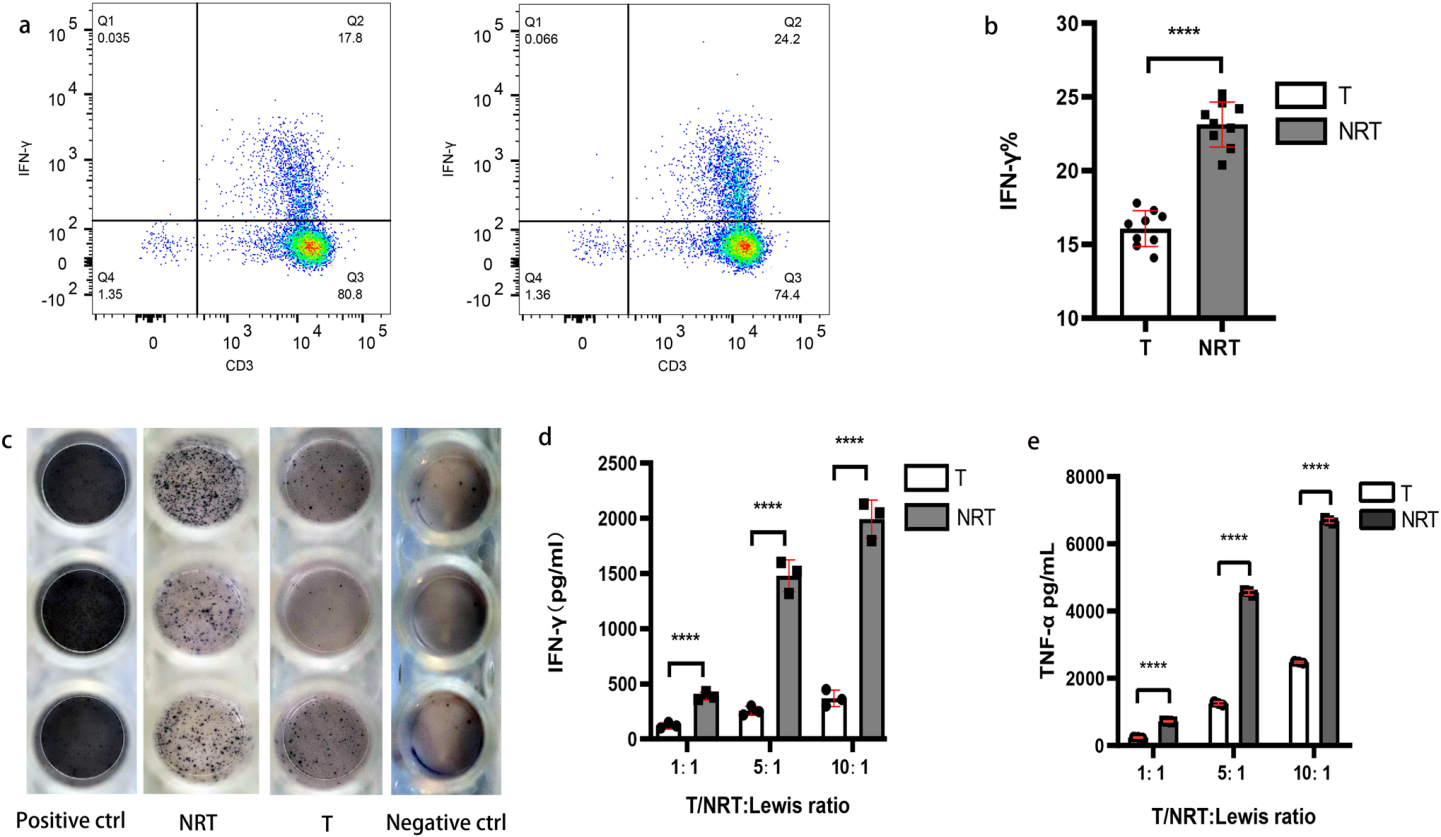
Functionality and immune response assessment of NRT cells: T cells were collected after REP. **a** and **b** Differences between IFN-γ secretion of conventional T cells and NRT cells following intracellular IFN-γ flow cytometry staining. **c** Images of the neoantigen-specific T-cell response after in vitro expansion of conventional T cells and NRT cells by ELISPOT assay. **d** and **e** Conventional T or NRT cells were co-cultured with Lewis cells in a 96-well plate for overnight. Cells were centrifuged, supernatant was collected, and the secreted IFN-γ and TNF-αlevels were determined through ELISA. *T* conventional T-cell group, *NRT* neoantigen-reactive T-cell group, *Positive ctrl* positive control, *Negative ctrl* negative control. Data shown are representative of three independent experiments. ****indicates that *P* < 0.0001

To produce large numbers of NRT cells for ACT, NRT cells were amplified using a 10–14-day rapid expansion protocol (REP), which resulted in the final drug product for transfusion. Before T-cell transfusion, the ability of NRT cells to specifically identify and mediate efficacy functions in response to Lewis tumour cells in vitro was evaluated through IFN-γ and TNF-α production and ELISpot assay. Cytokines, such as IFN-γ and TNF-α, contribute to the antitumor activity of cytotoxic T cells by inducing proliferative arrest and/or apoptosis (Vredevoogd et al. 2019). IFN-γ is a pro-inflammatory cytokine released from tumour suppressor cells, such as CD8^+^ cytotoxic T lymphocytes and CD4^+^ T cells, and suppresses angiogenesis in the tumour microenvironment (TME), kills pathogens, and stimulates adaptive immunity (Farhood et al. 2019). Flow cytometric staining indicated an increase in the IFN-γ levels from 17.8% in conventional T cells to 24.2% in NRT cells (Fig. 3a, b). This increase in IFN-γ levels can be attributed to the secretion of IFN-γ by more cells (higher percentage of cells producing IFN-γ), as well as to the large production of IFN-γ by the positive cells. Furthermore, as shown in Fig. 3c, the ELISpot assay results indicated that the immune reactivity of NRT cells were significantly augmented compared with that of conventional T cells, which is consistent with the results of IFN-γ and TNF-α level detected in the supernatant after conventional T or NRT cells were co-cultured with Lewis cells (Fig. 3d, e). The number and ability of T cells to secrete IFN-γ were strengthened after their stimulation by Neo-DCs. These three approaches could provide complementary and mutual corroborative information about NRT cells responses. Thus, our experiments confirmed that the NRT cells are proliferative and competent to stimulate in vitro antitumour immune response.

### Targeting neoantigens with ACT prevents Lewis tumour progression

**Fig. 4:**
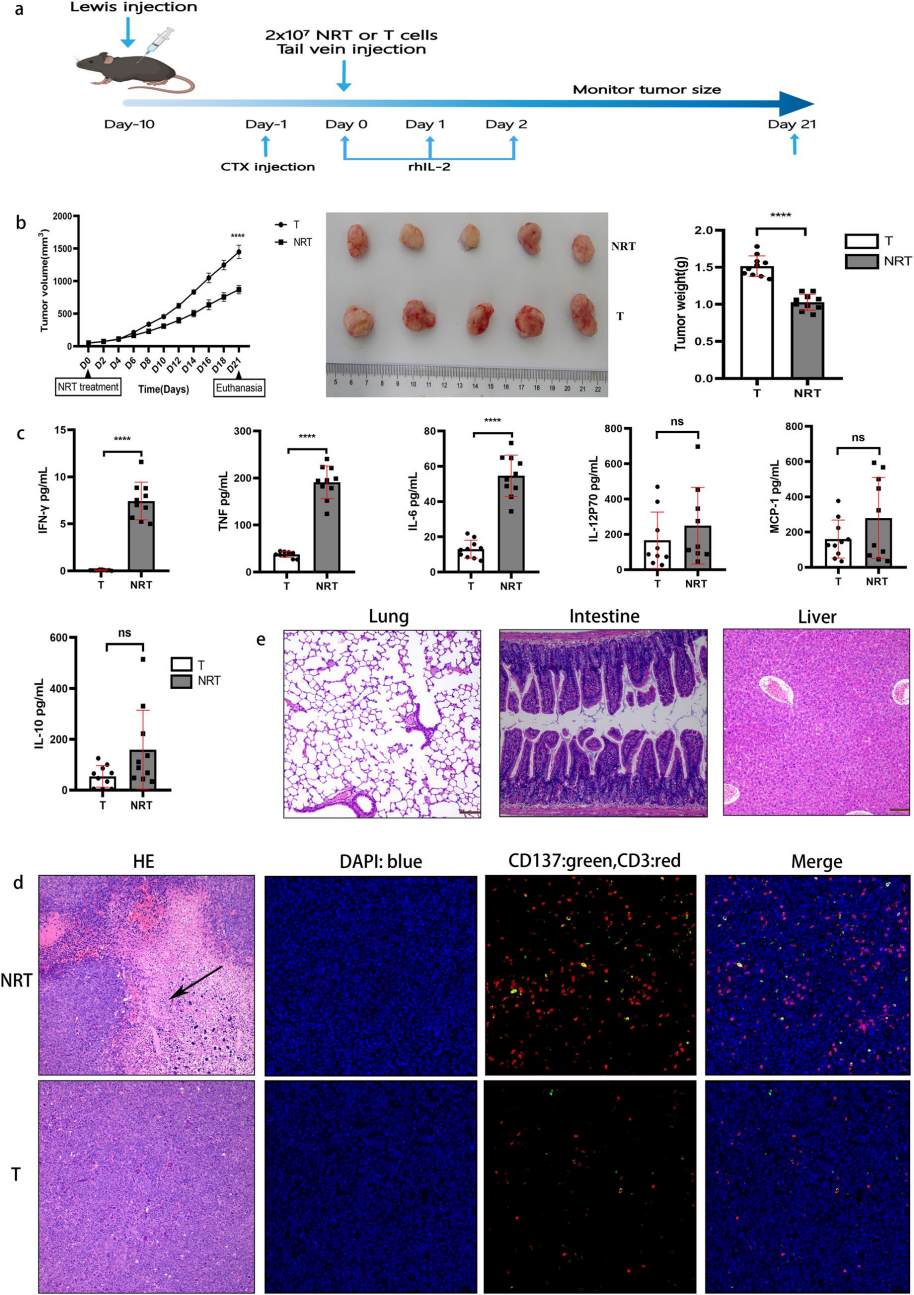
Antitumour immunity of NRT cells in vivo. **a** Lewis cells were transplanted into C57BL/6 mice. When tumours became palpable, the mice were randomised into two groups and received T cells or NRT cell treatment after CTX injection. rhIL-2 (180,000 U) was administereddaily for 3 days after NRT cell injection. All the mice were euthanised as indicated. **b** Line graphs showing that NRT cell injection significantly inhibited the tumour volume in tumour-bear mice (left). The middle and right graphs are illustrating that NRT cell injection remarkably suppressed the tumour volume and weight in the tumour-bearing mice. **c** CBA assay of inflammatory cytokines in the serum samples post-adoptive cell transfer of NRT cells. **d** Immunofluorescence analysis and histopathological evaluation of tumour tissues treated with NRT cells. On day 21, tumour tissues were resected from the mice and each tissue was divided into two parts. One part was stained with H&E (left) and the other part was used for immunofluorescence staining (IF, right). Necrotic regions in the tumours were observed in the mice treated with NRT cells (arrow indicates). In the IF analysis, a combination of anti-CD3 antibody (red) and anti-CD137 antibody (green) was used for primary staining. The nuclei were stained with DAPI (blue). Microscopic examination of IF samples was conducted at 400 × magnification. **e** H&E staining of lung, intestine, and liver of the mice who received NRT cells or T cells. Microscopic examination of H&E at 200 × magnification. *T* conventional T-cell group, *NRT* neoantigen-reactive T-cell group (*n* = 10). ****P < 0.0001. Results represent 1 of 2 independent experiments

Lung cancer neoantigens might be useful as targets for personalised cancer vaccines or other immunotherapeutic strategies. Hence, we attempted to elucidate whether the adoptive transfer of NRT cells isassociated with antitumour immunity in vivo. We established the Lewis tumour C57BL/6 mouse model and prepared NRT cells according to the aforementioned method. After tumours became palpable, 2 × 10^7^ NRT cells or conventional T cells were adoptively transferred to the tumour-bearing mice. As expected, the adoptive transfer of NRT cells was associated with a decreasing tumour growth, whereas the adoptive transfer of conventional T cells lead to arobust tumour growth rate in the mouse model (Fig. 4a, b). Our experiments thus indicated the therapeutic potential of NRT cells against Lewis tumours in vivo.

Recent studies have revealed that the increased levels of inflammatory cytokines, such as IFN-γ, TNF, and IL-6, in the serum result in a better response to NSCLC anti-PD-1 inhibition and prolonged survival, although these findings are debatable (Network 2012; Ozawa et al. 2019). In this study, we measured the IL-6, IL-10, MCP-1, IFN-γ, TNF and IL-12p7 protein levels in a single serum sample using the BD™ CBA Mouse Inflammation Kit. Our experiments demonstrated that the levels of all the six cytokines increased after NRT cell infusion, and the TNF, IFN-γ and IL-6 levels were significantly higher in NRT cell group than in the conventional T-cell group (Fig. 4c). This result suggests that multiple functions of NRT cells may capture the immunological signature relevant to the diversity of antitumour response. Furthermore, targeting neoantigens with ACT may potentially induce endogenous tumour-reactive T cells and their differentiation into multiple effector T lymphocytes.

Next, we performed immunofluorescence analysis and histopathological evaluation of the tumour in the treated mice. Necrotic regions in the tumours were observed in the mice treated with NRT cells but not in those treated with conventional T cells (Fig. 4d). In addition, the immunofluorescence analysis revealed that the treatment with NRT cells induces CD3^+^T-cell infiltration into the tumour tissue, which was substantially higher than in those treated with conventional T cells (Fig. 4d). Additionally, more infiltration of active T cells (CD3^+^ and CD37^+^T cell) into the tumour tissues and their co-localisation with CD3^+^T cells were observed after treatment with NRT cells than in conventional T cell-treated mice (Fig. 4d). Furthermore, treatment with NRT cells did not induce immune-related adverse events in other organs, such as lungs, intestine, and liver, during a limited observation period (Fig. 4e), indicating that the adoptive transfer of NRT cells is a low-toxicity therapy.

Most of the current immunotherapies focus on manipulating T cells, however, the tumor microenvironment (TME) is abundantly infiltrated by a heterogeneous population of tumor-associated macrophages (TAMs) and Foxp3 + regulatory T (Treg) cells, which are supppsed to be tumor-promoting immune cells and often associated with an increased resistance to cancer therapies (Mantovani et al. 2017; Tanaka and Sakaguchi 2017). To present the changes of the immune cells in TME after the NRT cell therapy, we investigated the infiltration of CD4 + /CD8 + T cells, TAMs and Treg cells in single-cell suspensions from primary mouse tumour tissue through flow cytometry. As expected, the CD4 + /CD8 + T cell percentage was obviously increased after NRT cell infusion (Figure S1a and b), suggesting an increase in the number of TILs infiltration into the tumour tissue. Meanwhile, there was no significant difference in the TAMs and Treg cells population between the two experiment groups (Figure S1c–f), indicating that the immuno-suppressor cells may not influence the anti-tumor effect of NRT therapy.

Collectively, we confirm from in vitro to in vivo experiments that the adoptive transfer of NRT cells can provoke the antitumour effect against mouse lung cancer and that this novel treatment strategy is feasible, safe, and effective.

## Discussion

The adoptive transfer of TILs can mediate the regression of metastatic solid tumours, and accumulating evidence suggests that therapeutic TILs target tumour-specific mutations (Chen et al. 2019; Dudley et al. 2002; Gros et al. 2016; Tran et al. 2014). In addition, adoptively transferred mutation-reactive T cells appear capable of mediating the rejection of metastatic epithelial cancers (Tran et al. 2014). The neoantigen burden in tumour tissue is directly and positively correlated with tumour mutation loads. The neoantigen burden has been reported to be significantly associated with the clinical outcomes of immunotherapy and patients’ prognosis. For cancers with high mutation loads (also possibly high neoantigen burden), such as melanoma or lung cancer, ICIs and neoantigen-based immunotherapy have been demonstrated to result in robust clinical responses. Among other solid tumours, lung cancer was chosen for this study because of an urgent unmet clinical need and the susceptibility of this cancer towards immunotherapy with PD-1/PDL-1 antibodies (Reck et al. 2016; Soria et al. 2018). In addition, lung cancer has the largest mutation load with the exception of melanoma (Rittmeyer et al. 2017). Since the mutated neo-antigenic peptides arising from tumour mutations are ideal targets for TILs (Li and Durbin 2009), lung cancer seems to be the optimal candidate for ACT.

As for the tumor vaccines, neoantigens can not only enhance the anti-tumor immune response, but also reduce the risk of autoimmunity to normal tissue. Based on the tumor neoantigens or mutant driving antigens, several types of tumor vaccines have been applied to clinical trials, including tumor cell vaccine (Inogés et al. 2017), synthetic long peptide (SLP) vaccine (Lilleby et al. 2017), nucleic acid vaccine (Sahin et al. 2017), and DC-based vaccine (Small et al. 2015). In 2017, Professor Ugur Sahin reported the first-in-human application of personalized RNA mutanome vaccines mobilize poly-specific therapeutic immunity against melanoma (Sahin et al. 2017). Recently, a case report suggested that a combination of neo-antigen peptide vaccination and NRT infusion is somewhat efficacious in arresting the development of duct carcinoma (CDC), which carries a low tumour mutation burden (Zeng et al. 2020). Another pilot study reported exact curative effect of personalised neo-antigen-pulsed DC vaccine in advanced lung cancer (Ding et al. 2021). With the deepening research of tumor vaccine and the preliminary results of clinical trials, the RNA vaccine demonstrated its unique advantages. First, RNA can be extracted from a small number of cancer cells and then amplified for vaccine preparation when adequate tumor tissue is not available. Second, RNA vaccine can avoid integration into host genome and latent risks are avoided compared with DNA vaccine. In this study, we used WES, RNA sequencing, and computational analysis such as silico analysis to identify and prioritise 10 potentially immunogenic somatic point mutations, followed by synthetic TMG as the template for generating of IVT RNA. The neo-antigenic-RNA vaccine was prepared through in vitro transcription, and then, the DC cells were transfected and co-cultured with tumour-bearing mouse T cells to obtain NRT cells. Some of these candidate neo-antigens were recognised by the immune system, thus presenting potential targets for cancer immunotherapy. We evaluated the T-cell activation proportion of NRT preparation through flow cytometry. The CD3 + /CD137 + -T cell percentage was increased from 0.072% in conventional T cells to 9.96% in NRT cells. After in vitro expansion, IFN-γ secretion augmented from 17.8% in conventional T cells to 24.2% in NRT cells and its immunoreactivity to tumour cells was significantly strengthened. As an in vivo adoptive therapy model, our animal experiments demonstrated that this treatment scheme can prevent tumour progression and exert a strong anti-tumour effect just as the displaying of pathological results. NRT cells promoted the infiltration of T cells (CD3 + T) and active T cells ((CD3 + /CD137 + T) into the tumour tissue, as well as their co-localisation. Most importantly, no irAE was found in the pathological staining of the lung, liver and intestine samples after the treatment. Hence, the strategy of using RNA vaccine to prepare NRT cells is feasible and effective.

Neoantigens are epitope peptides that bind to the MHC on the surface of malignant tumour cells and can be recognised by T cells, thus stimulating a strong specific anti-tumour immune response. Current research revealed that humans mount a mutation-specific T-cell response against epithelial cancers. Next-generation sequencing technologies (NGS) have provided insights into the biology and mutational landscape of cancer. With the development of NGS, the WES of the tumour DNA in combination with RNA-Seq and in silico HLA-binding prediction has been used to identify nonsynonymous cancer mutations recognised by T cells (Yin et al. 2019). Tumour neo-antigens can be predicted and identified according to the affinity analysis of peptides to MHC molecules or mass spectrometry analysis. A portion of mutational peptides can then be synthesised, pulsed onto the APCs, and tested for recognition by the autologous CD8/CD4^+^T lymphocytes. Immunotherapy based on the ACT of TILs has been shown to result in very good results in metastatic melanoma, cholangiocarcinoma, and colorectal cancer (Dudley et al. 2002; Tran et al. 2014, 2016). However, in clinical practice, sufficient tumour tissue samples are difficult to obtain from some patients because of their deteriorating physical conditions or tumour location. Such patients cannot benefit from the ACT treatment because of the amount of TILs isolated from the limited tissue samples is generally insufficient. Fortunately, a research group of Professor Cohen, C. J and Alena Gros confirmed that neoantigen-specific T cells can be isolated not only from tumour tissues but also from peripheral blood samples. Interestingly, circulating and tumour-infiltrating CD8^+^PD-1 + T cells share similar tumour-antigen specificities and TCR repertoires (Cohen et al. 2015; Gros et al. 2016). Importantly, neoantigen-specific T cells can also be identified in the peripheral blood samples of patients with gastrointestinal cancer, which is characterised by a low load of mutations (Gros et al. 2019). In the present study, T cells were isolated from the spleen of tumour-bearing mice to prepare NRT cells, and this strategy was found to be feasible, effective, and safe. In clinical practice, T cells can be collected through leukapheresis and co-cultured with Neo-DCs to prepare NRT cells, which can be used for treating patients with advanced solid tumours. This novel therapeutic strategy provides an opportunity to treat patients who cannot provide sufficient pathological specimens in a clinic and therefore cannot benefit from TIL-ACT treatment. Our study provides a rigorous scientific foundation for the clinical application of adoptive transfer of NRT cells.

Aspects, such as the number of neoantigens in Lewis cells and specific to which neoantigens, can exert their antitumour effect in vivo remain unclear, which may be a limitation of the present study. Although we identified somatic missense mutations through NGS, some mutations may not be expressed in practice. On the other hand, candidate neoantigens that are expressed and predicted to bind with a high affinity may not trigger detectable T-cell responses. In our next study, we aim to synthesise neoantigenic peptides, screen immunogenic-neoantigens for antitumour activity in vitro or with the help of mass spectrometry to more accurately filter the neoantigens and thus improve the antitumour effect of NRT cells. Moreover, the general biological traits of tumour and T cells, such as enhanced trafficking of T cells to solid tumour sites, overcoming the suppressive microenvironment, modulating T-cell senescence or exhaustion, and promoting proliferation and survival of T cells, can affect the antitumour effect (Kishton et al. 2017; Lim and June 2017). These obstacles remain to be overcome in further studies on the development of effective ACT immunotherapy.

## Conclusion

To conclude, NGS and epitope prediction strategies can identify and prioritise candidate neoantigens, and the NRT cells induced by RNA mutanome vaccine can elicit an antitumour reaction against mouse lung cancer. This study provides a basis for the adoptive transfer of NRT cells for lung cancer, which may serve as a feasible therapeutic approach for this disease.

## Supplementary Information

Below is the link to the electronic supplementary material.

Supplementary file1 Supplementary Fig. 1. The changes of the immune cells in TME after the ACT therapy: single tumour cells were collected from induced mouse tumour tissue. a and b The different T-cell percentage(CD4+/CD8+T-cell) in single tumour cell suspensions between conventional T cells(left) and NRT cells(right) group. c and d The number of TAMs(CD45+CD11b+F4/80+-cell) infiltration into the tumour tissue between conventional T cells(left) and NRT cells(right) group. e and f The different Treg cells percentage (CD4+FoxP3+T-cell) in single tumour cell suspensions between conventional T cells(left) and NRT cells(right) group. T: conventional T-cell group; NRT: neoantigen-reactive T-cell group. ***P <0.001, ****P <0.0001. Data are gated on single cells and further gated on live cells. (TIF 62515 kb)

Supplementary file2 Table S1.List of key materials and reagents (XLSX 14 kb)

## Data Availability

The data presented in this study are available in the article or supplementary files.
