# Supplementary figures and images for: Anti-tumour effect of neo-antigen-reactive T cells induced by RNA mutanome vaccine in mouse lung cancer
Source: J Cancer Res Clin Oncol. 2021 Jul 21;147(11):3255–68. doi: 10.1007/s00432-021-03735-y (PMC8484245; doi:10.1007/s00432-021-03735-y)

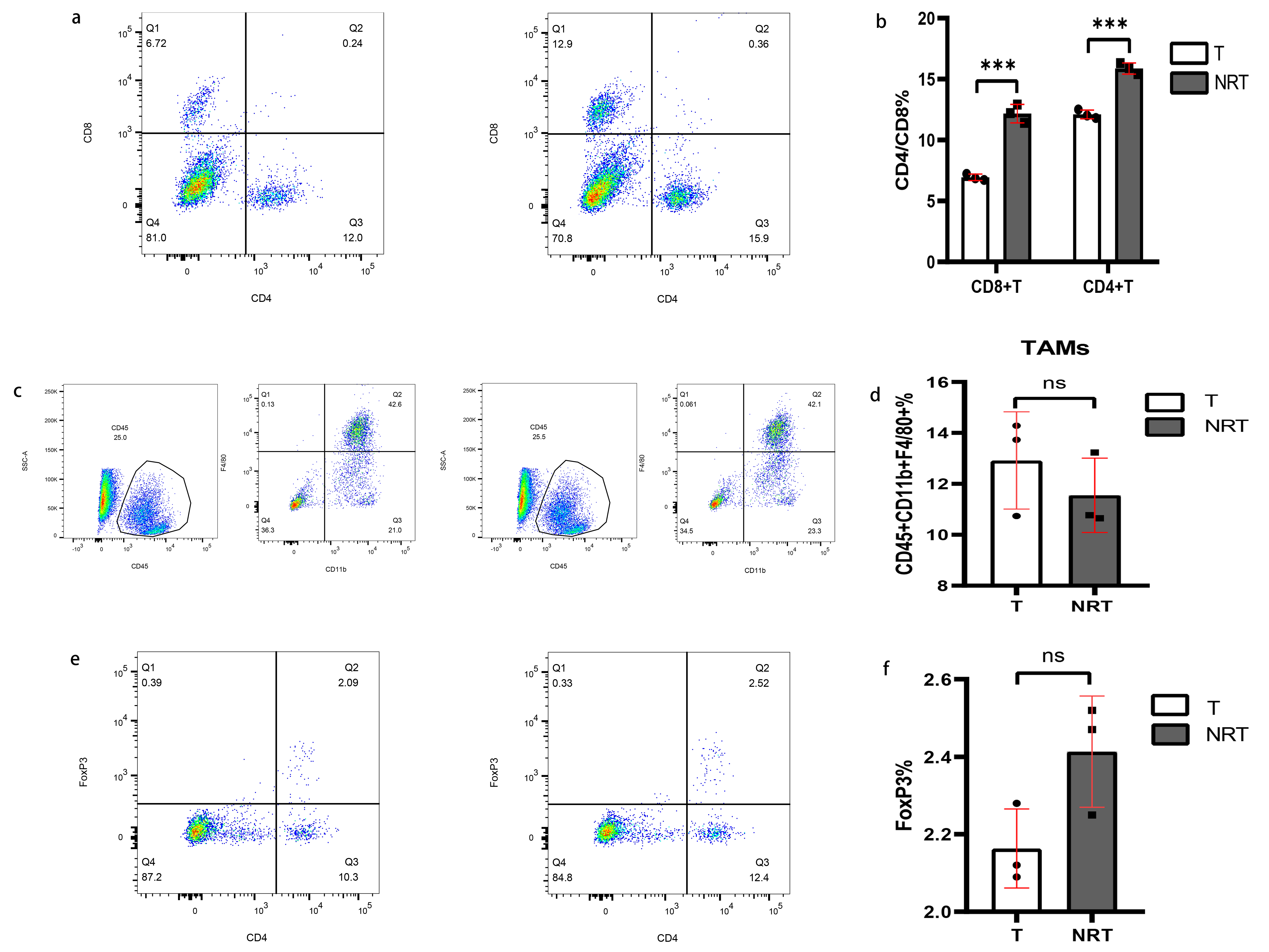

Supplement: Supplementary file 1 — Supplementary file1 Supplementary Fig. 1. The changes of the immune cells in TME after the ACT therapy: single tumour cells were collected from induced mouse tumour tissue. a and b The different T-cell percentage(CD4+/CD8+T-cell) in single tumour cell suspensions between conventional T cells(left) and NRT cells(right) group. c and d The number of TAMs(CD45+CD11b+F4/80+-cell) infiltration into the tumour tissue between conventional T cells(left) and NRT cells(right) group. e and f The different Treg cells percentage (CD4+FoxP3+T-cell) in single tumour cell suspensions between conventional T cells(left) and NRT cells(right) group. T: conventional T-cell group; NRT: neoantigen-reactive T-cell group. ***P <0.001, ****P <0.0001. Data are gated on single cells and further gated on live cells. (TIF 62515 kb) [file 432_2021_3735_MOESM1_ESM.tif]
